# Biofeedback-Assisted Rehabilitation for Neuromotor Recovery After Lower-Extremity Musculoskeletal Injury: A Narrative Review

**DOI:** 10.7759/cureus.113519

**Published:** 2026-07-28

**Authors:** Rasika Bhide, Harpreet Singh

**Affiliations:** 1 Orthopedics, Equipoise Physical Therapy and Wellness, Jersey City, USA; 2 Geriatrics, Select Rehabilitation, Folsom, USA

**Keywords:** athletic injuries, biofeedback, exercise therapy, neuromuscular training, proprioception, rehabilitation

## Abstract

Biofeedback delivers real-time physiological or biomechanical information during rehabilitation, but the postinjury evidence is heterogeneous and does not support a simple claim of superiority over exercise. This narrative review explored how biofeedback has been used to address neuromotor recovery after lower-extremity musculoskeletal injury and summarized direct exercise-only comparisons where available. PubMed/MEDLINE, Scopus, and PEDro were searched from inception through May 31, 2026, using database-specific combinations of terms for biofeedback, rehabilitation, anterior cruciate ligament injury or reconstruction, meniscectomy, chronic ankle instability, ankle sprain, and sports injury. Therapeutic primary studies were eligible when they enrolled injured or postoperative participants, used electromyographic, visual, or kinetic, force or pressure, auditory, tactile, or vibration feedback, and reported muscle activation, knee extension, proprioception, balance, gait, or loading, symmetry, movement quality, or functional outcomes. Healthy performance or prevention cohorts, exercise-only studies, assessment-only reports, protocols, conceptual papers, and secondary reviews were excluded from the core clinical synthesis; relevant secondary and healthy-cohort evidence was discussed separately. Sixteen primary clinical reports, comprising nine randomized trials and seven nonrandomized, acute, or uncontrolled studies, were included. Randomized trials were appraised against Physiotherapy Evidence Database domains and nonrandomized studies against the revised Joanna Briggs Institute quasi-experimental tool. Overall confidence was limited by small samples, incomplete allocation or blinding reporting, heterogeneous devices and dosing, task-specific outcomes, and short follow-up. Preliminary evidence suggests that electromyographic biofeedback may support early quadriceps activation or knee-extension recovery after anterior cruciate ligament reconstruction or meniscectomy, but pain and patient-reported function were not consistently superior to standard rehabilitation. Visual, kinetic, force-based, auditory, and vibration feedback produced task-specific changes in balance, strength, joint-position sense, gait, loading, or symmetry; retention and transfer were inconsistent, and a 2026 chronic ankle instability trial found no improvement in dynamic muscle-activation timing. Biofeedback, therefore, appears promising as a targeted adjunct when the feedback variable is matched to a defined impairment. Its additional benefit over well-designed exercise rehabilitation and its effects on return to sport and reinjury remain uncertain.

## Introduction and background

Musculoskeletal injury can alter more than tissue capacity. Even after pain, range of motion, and strength improve, an injured person may retain delayed muscle activation, inaccurate joint-position sense, altered loading, or inefficient task strategies. In this review, neuromotor control is used as the umbrella term for planning and executing coordinated movement through integrated sensory and motor processes. Neuromuscular control refers more specifically to muscle recruitment, timing, co-contraction, and force strategies that control a joint. Sensorimotor control describes the integration of sensory information with motor output; proprioception is the sense of joint position and movement; and movement quality is the observable alignment, sequence, symmetry, loading, and error pattern during a defined task [1-3].

Conventional rehabilitation remains the foundation of recovery and typically combines progressive range-of-motion work, strengthening, balance, and proprioceptive exercise, functional training, plyometrics, and graded sport exposure. Biofeedback is an adjunctive motor-learning strategy rather than a single treatment. It converts a physiological or biomechanical variable into information that a patient can use during a task [1,2]. Surface electromyographic biofeedback displays muscle activation; visual or kinetic systems display joint motion, ground-reaction force, balance, or symmetry; pressure systems display plantar loading; and auditory, tactile, or vibration cues signal whether a target has been reached. These technologies have different mechanisms and should not be interpreted as one interchangeable intervention.

Recent syntheses have examined electromyographic biofeedback after anterior cruciate ligament reconstruction and biomechanical biofeedback in chronic ankle instability [4,5]. However, the clinical literature remains distributed across different injuries, devices, training tasks, outcomes, and study designs. Earlier narratives also mixed postinjury rehabilitation with healthy performance or injury-prevention cohorts, making it difficult to determine what evidence applies to therapeutic recovery. A focused narrative synthesis is therefore useful if it separates injured populations from contextual healthy-cohort evidence, distinguishes between direct between-group effects and within-group change, and interprets each modality according to its specific feedback variable.

The objective of this narrative review was to explore the clinical use of biofeedback for neuromotor recovery after lower-extremity musculoskeletal injury, with emphasis on anterior cruciate ligament injury or reconstruction, meniscal surgery, and chronic ankle instability. Direct comparisons with exercise-only rehabilitation were summarized when available, but the review was not designed as a formal comparative-effectiveness analysis.

## Review

Materials and methods

Review Design and Reporting Approach

This paper was retained as a narrative review because the evidence spans randomized trials, nonrandomized controlled studies, uncontrolled clinical series, and acute mechanistic experiments with substantial clinical and technological heterogeneity. The revised structure follows the Scale for the Assessment of Narrative Review Articles (SANRA), particularly its requirements for a clear rationale, a focused objective, a described literature search, appropriate referencing, scientific reasoning, and presentation of relevant endpoint data [6]. The review was not converted into a systematic review or meta-analysis, and Preferred Reporting Items for Systematic Reviews and Meta-Analyses (PRISMA)-based pooled or vote-counting displays were not used.

Search Strategy

PubMed/MEDLINE, Scopus, and PEDro were searched from database inception through May 31, 2026. Database-specific strategies are shown in Table 1. Reference lists of eligible reports and relevant reviews were also examined. The search was limited to English-language human studies. Duplicate citations were identified using title, first author, year, and digital object identifier before screening.

**Table 1:** Database-specific search strategies.

Database	Search strategy
PubMed/MEDLINE	("biofeedback"[Title/Abstract] OR "electromyographic biofeedback"[Title/Abstract] OR "EMG biofeedback"[Title/Abstract] OR "visual feedback"[Title/Abstract] OR "auditory feedback"[Title/Abstract] OR "haptic feedback"[Title/Abstract] OR "kinematic feedback"[Title/Abstract] OR "gait retraining"[Title/Abstract]) AND (rehabilitation[Title/Abstract] OR exercise[Title/Abstract] OR training[Title/Abstract]) AND ("anterior cruciate ligament"[Title/Abstract] OR menisc*[Title/Abstract] OR "chronic ankle instability"[Title/Abstract] OR "ankle sprain"[Title/Abstract] OR "musculoskeletal injury"[Title/Abstract] OR athlete*[Title/Abstract])
Scopus	TITLE-ABS-KEY ( biofeedback OR "electromyographic biofeedback" OR "visual feedback" OR "auditory feedback" OR "haptic feedback" OR "kinematic feedback" OR "gait retraining" ) AND TITLE-ABS-KEY ( rehabilitation OR exercise OR training ) AND TITLE-ABS-KEY ( athlete* OR "anterior cruciate ligament" OR menisc* OR "chronic ankle instability" OR "ankle sprain" OR "musculoskeletal injury" )
PEDro	The term "biofeedback" was paired separately with "anterior cruciate ligament," "ACL reconstruction," "meniscectomy," and "chronic ankle instability."

Eligibility Criteria

Primary clinical studies were eligible when they: (1) enrolled people with a lower-extremity musculoskeletal injury, a postoperative state, or chronic ankle instability; (2) applied biofeedback therapeutically during rehabilitation or gait/movement retraining; (3) used electromyographic, visual or kinetic, force or pressure, auditory, tactile, haptic, or vibration feedback; and (4) reported a neuromotor outcome such as muscle activation or timing, active knee extension, proprioception or joint-position error, balance or postural control, gait or plantar loading, movement quality, symmetry, or functional performance.

Studies were excluded from the core clinical synthesis when they enrolled only healthy or uninjured participants, addressed primary injury prevention or general performance, evaluated exercise without a biofeedback component, used biofeedback only for diagnosis or assessment, focused on neurological rehabilitation, or were protocols, conceptual frameworks, conference summaries, reviews, or meta-analyses. Relevant systematic reviews were used to contextualize the primary evidence, and one recent healthy-cohort randomized trial was described separately because it was specifically raised during peer review; neither was counted as postinjury clinical evidence.

Study Selection and Data Extraction

RB performed the electronic search, duplicate removal, and initial title-and-abstract screening. HS independently verified full-text eligibility and extracted study characteristics and outcomes; both authors checked the final evidence tables and methodological judgments. Disagreements were resolved through discussion. Extracted variables were author and year, design, sample, and injury, feedback modality, comparator, intervention duration, outcomes, between-group and within-group findings, retention or transfer testing, and principal methodological limitations. Because the original record-level export and exclusion log were not available in the reviewer file, screening counts were not retrospectively reconstructed. The final included primary reports are listed transparently in Table 2 rather than displayed in a PRISMA flow diagram.

**Table 2:** Primary clinical evidence on biofeedback-assisted rehabilitation. ACL, anterior cruciate ligament; EMG, electromyography

Study	Design and population	Feedback/comparator	Neuromotor outcomes	Main findings	Methodological cautions
Draper (1990) [9]	Randomized trial; *n* = 22 after ACL reconstruction	EMG biofeedback plus exercise vs. exercise alone	Active knee extension; quadriceps isometric peak torque	Biofeedback-facilitated exercise accelerated recovery of full active extension and improved later quadriceps torque relative to exercise alone.	Small older trial; allocation concealment and assessor blinding were not clearly reported; rehabilitation protocols predate current practice.
Christanell et al. (2012) [10]	Randomized trial; *n* = 16, early after ACL reconstruction	EMG biofeedback plus standard therapy vs. standard therapy	Passive knee extension; handheld dynamometry; vastus medialis EMG; pain/function	Between-group findings favored biofeedback for knee-extension recovery and selected strength/activation measures; pain and functional outcomes were not consistently different.	Very small sample; short six-week follow-up; participant and therapist blinding not feasible.
Akkaya et al. (2012) [11]	Single-blind randomized trial; *n* = 45 after arthroscopic partial meniscectomy	EMG biofeedback plus exercise vs. electrical stimulation plus exercise vs. home exercise	Quadriceps activation; gait milestones; Lysholm score; strength	EMG biofeedback improved selected quadriceps activation and early rehabilitation milestones relative to comparison groups, particularly at the second postoperative week.	Three small groups; short-term outcomes; multiple comparisons and limited longer-term follow-up.
Morales-Sanchez et al. (2022) [12]	Uncontrolled clinical series; *n* = 10 professional soccer players after meniscectomy	Quadriceps EMG biofeedback added to rehabilitation; no control group	Vastus lateralis/medialis activation and reliability	Quadriceps EMG activity improved across treatment sessions, supporting feasibility in athletes.	No comparator, very small sample, and no basis for attributing change specifically to biofeedback.
Kumari and Kakkad (2025) [13]	Nonrandomized controlled pilot; *n* = 12 with quadriceps lag after ACL reconstruction	Real-time visual biofeedback plus rehabilitation vs. standard rehabilitation	Quadriceps lag; active knee-extension range of motion	Both groups improved; the biofeedback group showed greater short-term improvement in quadriceps lag and active extension.	Nonrandom allocation, six participants per group, two-week intervention, and no durability or return-to-sport outcomes.
Molka et al. (2015) [14]	Prospective pre-post study; *n* = 16 after ACL reconstruction plus 15 healthy controls	One-month visual balance biofeedback program; no injured nonfeedback comparator	Static/dynamic balance; Lysholm score	Selected balance measures improved after training and approached healthy-control values.	Healthy controls were not an active rehabilitation comparator; improvement may reflect time and concurrent rehabilitation.
Luc-Harkey et al. (2018) [15]	Acute repeated-measures laboratory study after ACL reconstruction	Real-time visual force biofeedback during walking	Vertical ground-reaction force; knee flexion excursion; knee extension moment	Participants could increase or decrease targeted gait variables during the feedback session.	Demonstrates immediate controllability, not retained recovery; no exercise-only group or clinical follow-up.
Emami Meibodi et al. (2022) [16]	Randomized clinical trial; *n* = 30 with ACL injury	Balance rehabilitation with visual feedback vs. the same program without feedback.	Knee joint-position error at 45 and 90 degrees; Berg Balance Scale	Between-group differences favored visual feedback for joint-position error. Balance improved within both groups.	Small sample; limited reporting of concealment/blinding; short four-week follow-up.
Peebles et al. (2022) [17]	Phase I randomized clinical trial; *n* = 40 after ACL reconstruction	Visual and tactile squat/landing feedback vs. control rehabilitation	Vertical ground-reaction-force symmetry; knee-moment symmetry; retention	Vertical ground-reaction-force symmetry improved immediately, but retention was limited; knee-moment symmetry did not show a significant group-by-time effect.	Phase I sample, task-specific endpoints, and no reinjury or return-to-sport outcome.
Ge et al. (2026) [18]	Randomized trial; 42 assigned, 33 completed after ACL reconstruction	Visual-feedback balance training plus rehabilitation vs. rehabilitation alone	Knee strength, dynamic balance, functional scores	The visual-feedback group showed greater improvements in selected strength, dynamic balance, and functional outcomes after ten weeks.	Small completer sample, attrition, short follow-up, retrospective registration, and potential device/task learning effects.
Donovan et al. (2016) [19]	Acute within-subject study in chronic ankle instability	Auditory plantar-pressure biofeedback during walking	Regional plantar pressure; muscle activation	Feedback reduced lateral plantar loading and altered selected muscle-activation measures during walking.	Immediate laboratory response only; no active comparator, retention test, or patient-reported outcome.
Torp et al. (2019) [20]	Acute within-subject study; *n* = 26 with chronic ankle instability	Real-time visual feedback during treadmill walking	Plantar pressure and center-of-pressure location	Visual feedback shifted loading medially and reduced lateral plantar pressure during the session.	No control group or follow-up; clinical importance and transfer to unrestricted activity were not established.
Koldenhoven et al. (2021) [21]	Randomized trial; *n* = 27 with chronic ankle instability	Visual gait biofeedback plus impairment-based rehabilitation vs. the same rehabilitation without feedback	Walking biomechanics; patient-reported function; impairment measures	Both groups improved, while feedback produced additional changes in targeted gait biomechanics and selected patient-reported outcomes.	Small sample, limited blinding, and short four-week intervention; durability beyond the study window was uncertain.
Migel and Wikstrom (2021) [22]	Repeated-measures study; *n* = 19 with chronic ankle instability	Laboratory and real-world gait training using vibration feedback	Center-of-pressure trajectory during gait; short retention	Vibration feedback shifted the center-of-pressure location medially, with short-term retention after training.	No control group; retention was brief, and clinical outcomes were not evaluated.
Torp et al. (2022) [23]	Randomized controlled trial; *n* = 18 with chronic ankle instability	Auditory gait biofeedback vs. no-feedback control	Plantar pressure/force; center of pressure; talar cartilage characteristics	After eight sessions, feedback reduced lateral loading and produced a medial center-of-pressure shift that persisted at one week; talar cartilage measures did not change.	Small and imbalanced groups, short intervention and follow-up, and no recurrence or long-term function outcome.
Shamsi et al. (2026) [24]	Three-arm randomized trial; 45 randomized, 39 analyzed; elite athletes with chronic ankle instability	EMG biofeedback vs. neurofeedback vs. control	Time to peak and amplitude of gastrocnemius, tibialis anterior, and peroneus longus EMG during jump/hop tasks	Neither EMG biofeedback nor neurofeedback produced significant within-group or between-group improvement in dynamic muscle-activation timing or amplitude.	Small groups, isolated training tasks, incomplete blinding, and possible mismatch between training task and dynamic test outcomes.

Methodological Appraisal

Randomized trials were appraised against PEDro domains, including random allocation, concealment, baseline comparability, blinding where feasible, follow-up, intention-to-treat analysis, between-group comparisons, and reporting of point estimates and variability [7]. Nonrandomized, acute, and uncontrolled studies were appraised using domains from the revised Joanna Briggs Institute tool for quasi-experimental studies, including temporal clarity, group comparability, presence of a comparator, repeated measurement, follow-up, outcome reliability, and appropriateness of statistical analysis [8]. Because blinding of participants and therapists is often impractical in feedback-based rehabilitation and several reports incompletely described appraisal domains, no pooled quality score was calculated. Item-level concerns were used to determine interpretive weight and are summarized in Table 3.

**Table 3:** Structured methodological appraisal and interpretive weight. PEDro, Physiotherapy Evidence Database; JBI, Joanna Briggs Institute; EMG, electromyography

Study	Appraisal framework	Principal strengths	Principal concerns and interpretive weight
Draper (1990) [9]	PEDro domains	Randomized exercise comparator; clinically relevant extension and torque outcomes.	Small older trial; concealment, assessor blinding, and contemporary protocol equivalence unclear. Interpret as preliminary direct-comparison evidence.
Christanell et al. (2012) [10]	PEDro domains	Randomized comparator; between-group testing; objective extension, force, and EMG outcomes.	n = 16; short follow-up; limited concealment/blinding reporting. Low-to-moderate confidence.
Akkaya et al. (2012) [11]	PEDro domains	Randomized, three-group, single-blind design; objective and functional outcomes.	Small groups and short-term multiplicity. Moderate confidence for early recovery only.
Morales-Sanchez et al. (2022) [12]	JBI quasi-experimental domains	Repeated measurements and directly measured EMG response.	No comparator and n = 10. Very low confidence for causal efficacy.
Kumari and Kakkad, 2025) [13]	JBI quasi-experimental domains	Concurrent standard-rehabilitation comparator; clinically understandable endpoints.	Nonrandom allocation, n = 12, two weeks, no blinded assessment or follow-up. Low confidence.
Molka et al. (2015) [14]	JBI quasi-experimental domains	Prospective assessment, objective balance testing; healthy reference group.	No injured no-feedback comparator. Low confidence in the added effect of feedback.
Luc-Harkey et al. (2018) [15]	JBI quasi-experimental domains	Controlled within-session manipulation of specified gait variables.	Acute mechanistic design; no retention or clinical comparator. Useful for feasibility, not efficacy.
Emami Meibodi et al. (2022) [16]	PEDro domains	Randomized same-exercise comparator; explicit between-group proprioception findings.	Small sample, brief follow-up, and limited allocation/blinding information. Moderate confidence for short-term joint-position error.
Peebles et al. (2022) [17]	PEDro domains	Randomized phase I design; objective biomechanics and retention testing.	Phase I sample; mixed retained effects; no clinical event outcomes. Moderate confidence for immediate task response.
Ge et al. (2026) [18]	PEDro domains	Randomized active comparator; multiple strength, balance, and function outcomes.	Attrition, small completer sample, retrospective registration, and short follow-up. Moderate but not definitive confidence.
Donovan et al. (2016) [19]	JBI quasi-experimental domains	Objective pressure and muscle measures under a defined cue.	Acute uncontrolled response. Very low confidence for sustained clinical benefit.
Torp et al. (2019) [20]	JBI quasi-experimental domains	Objective plantar-pressure outcomes and standardized tasks.	Acute no-control design without retention. Mechanistic evidence only.
Koldenhoven et al. (2021) [21]	PEDro domains	Randomized active comparator; impairment, gait, and patient-reported outcomes.	n = 27 and limited blinding/long-term follow-up. Moderate confidence for short-term targeted gait effects.
Migel and Wikstrom, 2021) [22]	JBI quasi-experimental domains	Laboratory and real-world feedback exposure with short retention testing.	No comparator and n = 19; retention measured only briefly. Low confidence.
Torp et al. (2022) [23]	PEDro domains	Randomized control; repeated gait training; one-week retention and cartilage outcome.	n = 18 with imbalanced groups; short follow-up. Moderate-to-low confidence.
Shamsi et al. (2026) [24]	PEDro domains	Randomized three-arm design and dynamic EMG outcomes.	Small groups, possible task-outcome mismatch, limited blinding, and no functional follow-up. Moderate confidence in the null finding for the measured outcomes.

Narrative Synthesis

Findings were synthesized narratively by anatomical region and feedback mechanism. Electromyographic feedback was interpreted separately from visual or kinetic, force or pressure, auditory, tactile, and vibration feedback. Direct between-group differences were distinguished from within-group improvement, and immediate task modification was distinguished from retained or transferred clinical change. No meta-analysis, pooled percentages, study-level win/loss classification, or frequency chart was produced because such summaries would give equal weight to studies with different designs, sample sizes, comparators, and outcome measures.

Findings

Study Characteristics

Sixteen primary clinical reports met the revised core eligibility criteria [9-24]. Ten addressed anterior cruciate ligament injury or reconstruction or meniscal surgery, and six addressed chronic ankle instability. Nine were randomized trials, and seven were nonrandomized controlled, uncontrolled, or acute within-subject studies. No eligible upper-extremity study was identified; accordingly, the title and clinical scope were narrowed to lower-extremity injury. Table 2 summarizes the primary evidence, and Table 3 presents the structured methodological appraisal.

Electromyographic Biofeedback

Electromyographic biofeedback provides direct information about the magnitude or timing of muscle activation and is most relevant when the clinical target is voluntary recruitment rather than whole-task alignment. After anterior cruciate ligament reconstruction, two small randomized trials reported faster knee-extension recovery and selected gains in quadriceps torque or activation when electromyographic biofeedback was added to exercise [9,10]. After partial meniscectomy, a three-group randomized trial reported earlier gains in selected quadriceps activation and rehabilitation milestones with electromyographic biofeedback [11]. These findings are clinically plausible for early arthrogenic muscle inhibition, but the samples were small, and pain or patient-reported function was not consistently superior.

The remaining electromyographic evidence is less definitive. An uncontrolled series of ten professional soccer players showed increasing quadriceps activity during post-meniscectomy rehabilitation, but the absence of a comparator prevents causal attribution [12]. A 2025 nonrandomized pilot of real-time visual biofeedback for quadriceps lag included only six participants per group and reported short-term improvement, but it did not establish durable superiority over standard rehabilitation [13]. In chronic ankle instability, a 2026 randomized trial found no significant improvement in dynamic lower-leg muscle timing or amplitude after electromyographic biofeedback, highlighting that feedback effects may not transfer when the training task and performance test differ [24].

Visual, Kinetic, Force-Based, and Tactile Feedback After Knee Injury or Surgery

Visual, kinetic, force-based, and tactile systems display movement or loading errors rather than muscle activity. A one-month visual-balance program after anterior cruciate ligament reconstruction improved selected balance measures, but it lacked an injured no-feedback comparator [14]. In a laboratory walking study, participants after reconstruction could deliberately increase or decrease vertical ground-reaction force, knee excursion, and knee moment in response to real-time visual cues; this demonstrated immediate controllability rather than retained clinical recovery [15].

Two randomized studies provide more direct exercise-controlled evidence. In people with anterior cruciate ligament injury, adding visual feedback to the same balance program reduced joint-position error more than balance exercise without feedback, whereas balance scores improved within both groups [16]. In a phase I post-reconstruction trial, visual and tactile feedback improved vertical ground-reaction-force symmetry immediately, but retention was incomplete, and knee-moment symmetry did not show a significant group-by-time effect [17]. A 2026 randomized trial reported greater gains in selected strength, dynamic balance, and function after visual-feedback balance training than after rehabilitation alone; interpretation remains cautious because only 33 participants completed the study, follow-up was short, and registration was retrospective [18].

Auditory, Plantar Pressure, and Vibration Feedback in Chronic Ankle Instability

In chronic ankle instability, feedback has most often targeted lateral plantar loading and center-of-pressure location. Acute auditory and visual feedback studies showed that participants could shift loading medially during treadmill walking [19,20]. These experiments support the feasibility of modifying a defined gait variable, but they do not demonstrate retained benefit or fewer recurrent sprains.

Repeated-training studies provide limited evidence of short-term retention. A four-week randomized trial found that visual gait biofeedback added to impairment-based rehabilitation produced additional changes in targeted gait biomechanics and selected patient-reported outcomes compared with the same rehabilitation without feedback [21]. Vibration feedback used in laboratory and real-world walking produced a medial center-of-pressure shift with brief retention, but the study lacked a control group [22]. In a small randomized trial, eight sessions of auditory gait feedback reduced lateral plantar loading and retained a medial center-of-pressure shift at one week; talar cartilage characteristics did not change [23]. Across these studies, the feedback variable was biomechanical and task-specific. The evidence, therefore, supports short-term gait modification more strongly than broad claims about joint stability, return to sport, or reinjury prevention.

Secondary and Contextual Evidence

The 2024 systematic review of electromyographic biofeedback after anterior cruciate ligament reconstruction reported pooled signals for selected knee-extension, strength, or balance outcomes, but not consistent superiority for pain or Lysholm function [4]. The chronic ankle instability meta-analysis similarly supported changes in center-of-pressure and lateral-loading variables while emphasizing heterogeneity [5]. These reviews are consistent with the present interpretation that biofeedback can change a specified target without necessarily improving every clinical outcome.

A 2025 randomized trial of neuromuscular training with real-time biomechanical biofeedback in healthy adolescent female soccer players was added as contextual evidence because it was identified during peer review [25]. It was not included in the core postinjury synthesis because participants were healthy, and the purpose was prevention or performance, not therapeutic rehabilitation. Table 4 separates this study and the two systematic reviews from the clinical primary evidence.

**Table 4:** Secondary and contextual evidence kept separate from the core clinical synthesis. ACL, anterior cruciate ligament

Source	Evidence type/population	Contribution	Role in this review
Ananias et al. (2024) [4]	Systematic review and meta-analysis after ACL reconstruction	Synthesized EMG-biofeedback trials and reported signals for knee extension, quadriceps recovery, and balance, while pain and Lysholm outcomes were not consistently different.	Secondary evidence; used to contextualize, not counted as a primary study.
Mousavi et al. (2023) [5]	Systematic review and meta-analysis of chronic ankle instability	Reported that biofeedback can modify center-of-pressure and lateral-loading biomechanics, with substantial modality and study heterogeneity.	Secondary evidence supports biomechanical plausibility but not uniform clinical efficacy.
Ford et al. (2025) [25]	Randomized trial in healthy adolescent female soccer athletes	Examined neuromuscular training with real-time biomechanical biofeedback in an injury-prevention/performance setting.	Healthy cohort; intentionally excluded from the core postinjury synthesis and presented only as contextual evidence.

Discussion

The revised evidence base does not support a binary conclusion that biofeedback is superior or inferior to traditional exercise. Conventional exercise remains the common foundation in almost every controlled study. Biofeedback adds information about a selected variable, and its value depends on whether that variable corresponds to a modifiable impairment, whether the patient can use the cue, and whether the new strategy persists after feedback is removed.

The modality distinction is clinically important. Electromyographic feedback is a physiological cue that can help a patient detect and increase voluntary muscle recruitment; its most plausible role is early quadriceps re-education when activation failure or extension lag is measurable. Visual force, kinematic, or balance feedback is biomechanical error information and is better suited to gait loading, symmetry, postural control, or movement alignment. Auditory, tactile, and vibration systems can signal threshold errors without requiring continuous visual attention, which may help practice outside a laboratory. These modalities should not be pooled as if they share the same mechanism, dose, or outcome.

The most consistent pattern was task specificity. Participants often changed the exact variable displayed to them during the intervention, including quadriceps activation, knee-extension range, joint-position error, ground-reaction-force symmetry, plantar pressure, or center-of-pressure location. The transfer was less certain. Immediate gait or landing changes were not always retained, and improvements in one symmetry metric did not guarantee improvement in another. The null 2026 chronic ankle instability trial further suggests that isolated biofeedback practice may not alter muscle timing during more complex jump or hop tasks when the training and outcome contexts differ [24].

For clinical use, feedback should therefore be prescribed as a targeted adjunct rather than a device-led treatment. A practical sequence is to define the impairment, choose a valid measurable signal, set an achievable target, practice with feedback, progressively reduce cue frequency, and retest the task without feedback and under more demanding conditions. Standard strengthening, range-of-motion work, balance exercise, graded loading, and sport-specific exposure should continue. A favorable response within a feedback session should not be interpreted as proof of long-term recovery.

Direct evidence of additional benefit over well-designed exercise was limited and mixed. Some small randomized trials reported between-group advantages for knee extension, quadriceps activation, proprioceptive error, selected balance or strength outcomes, or targeted gait biomechanics [9-11,16,18,21,23]. Other studies showed only within-group improvement, immediate task modification, incomplete retention, or no significant between-group effect [12-15,17,19,20,22,24]. Return-to-sport readiness, time to return, recurrent injury, and long-term participation were rarely reported. These gaps prevent a broad comparative-effectiveness conclusion.

Limitations

This review has limitations. It is a narrative rather than a systematic review, was restricted to English-language reports, and did not include grey literature. Although database-specific strategies and eligibility criteria are now reported, the original record-level export and screening log were not available, so a retrospective flow diagram and verified exclusion counts could not be produced. The search endpoint and two-author workflow should be checked against the authors' original records before final submission.

The evidence itself is heterogeneous. Injury stage, age, athletic status, feedback device, signal processing, cue schedule, treatment dose, exercise comparator, and outcome measures varied substantially. Many reports enrolled fewer than 30 participants, assessor blinding and allocation concealment were frequently unclear, and follow-up was commonly limited to the intervention period or several days. Acute laboratory studies established modifiability but not clinical effectiveness. The review also focuses on knee and ankle conditions; its conclusions should not be generalized to upper-extremity rehabilitation.

Finally, the structured PEDro- and Joanna Briggs Institute-based appraisal was qualitative. It was used to moderate interpretation rather than to generate a pooled quality score. No meta-analysis was attempted because clinical and methodological heterogeneity made a common effect estimate inappropriate. The removed percentage figures would have given identical weight to randomized trials, uncontrolled series, and conceptual evidence and therefore were not retained.

## Conclusions

Biofeedback may be a useful targeted adjunct to lower-extremity musculoskeletal rehabilitation when the measured signal is matched to a clearly defined neuromotor impairment. Preliminary evidence is most supportive of early quadriceps activation or knee-extension work after anterior cruciate ligament reconstruction or meniscal surgery and of task-specific modification of balance, gait loading, plantar pressure, or symmetry. However, effects are not uniform across modalities or outcomes, and immediate or within-group improvement does not establish superiority over standard exercise. The additional benefit of biofeedback over well-designed rehabilitation, as well as its influence on return to sport and reinjury, remains uncertain. Future studies should use adequately powered and prospectively registered randomized designs, active exercise comparators, standardized feedback dosing, blinded outcome assessment when feasible, and explicit tests of retention, transfer, return to sport, and recurrent injury.
